# Forest Health Status in North America

**DOI:** 10.1100/tsw.2007.85

**Published:** 2007-03-21

**Authors:** Borys Tkacz, Ben Moody, Jaime Villa Castillo

**Affiliations:** ^1^ USDA Forest Service, Forest Health Protection, Arlington, VA, USA; ^2^ Canadian Forest Service, Ottawa, Canada; ^3^ Comision Nacional Forestal, Zapopan, Jalisco, Mexico

**Keywords:** forest health monitoring, forest fires, forest pests, air pollution

## Abstract

The forests of North America provide a variety of benefits including water, recreation, wildlife habitat, timber, and other forest products. However, they continue to face many biotic and abiotic stressors including fires, native and invasive pests, fragmentation, and air pollution. Forest health specialists have been monitoring the health of forests for many years. This paper highlights some of the most damaging forest stressors affecting North American forests in recent years and provides some projections of future risks.

## FORESTS OF NORTH AMERICA

The forests of North America provide a variety of benefits including water, recreation, wildlife habitat, timber, and other forest products. Forests cover 677.5 million ha or nearly one-third of the total land area of North America[1]. This represents over 17% of the total global forest area, with Canada and the U.S. ranking third and fourth, respectively, among countries with the largest forest area. The total area of forests in Canada, Mexico, and the U.S. has been relatively stable over the last 15 years (Table 1).

**TABLE 1**
**Total Forest Area (million ha) by Country and Year[1]**

| Year | Canada | Mexico | U.S. |
|---|---|---|---|
| 1990 | 310 | 69 | 298.6 |
| 2000 | 310 | 65.5 | 302.3 |
| 2005 | 310 | 64.2 | 303 |

Although the total forest area in the U.S. has been relatively stable, some portions of the country have been increasingly fragmented. Fragmentation of forests may lead to changes in ecological processes and reduce biological diversity. Analyses of forestland maps derived from satellite imagery indicate that large portions of the forestland are fragmented with about 44% being within 90 m of the forest edge[2].

## FOREST FIRES

Fire is a major disturbance agent in many forests of North America. The amount of forest area burned varies depending on weather conditions, fuel loading, and forest stand conditions[2,3,4]. The total area of forest fires by country and year are presented in Figs. 1, 2, and 3. Many years of fire suppression have resulted in increased fuel loads and dense forests, resulting in increased risks of catastrophic, stand-replacing fires.

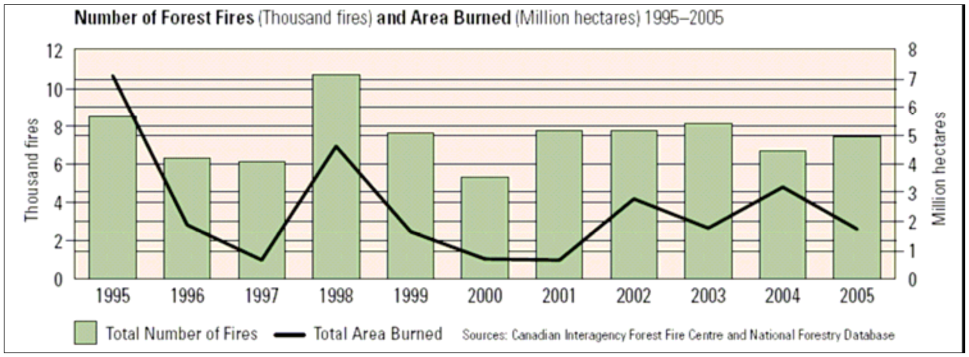

**FIGURE 1**. Historic trend in forest fires for Canada[3].

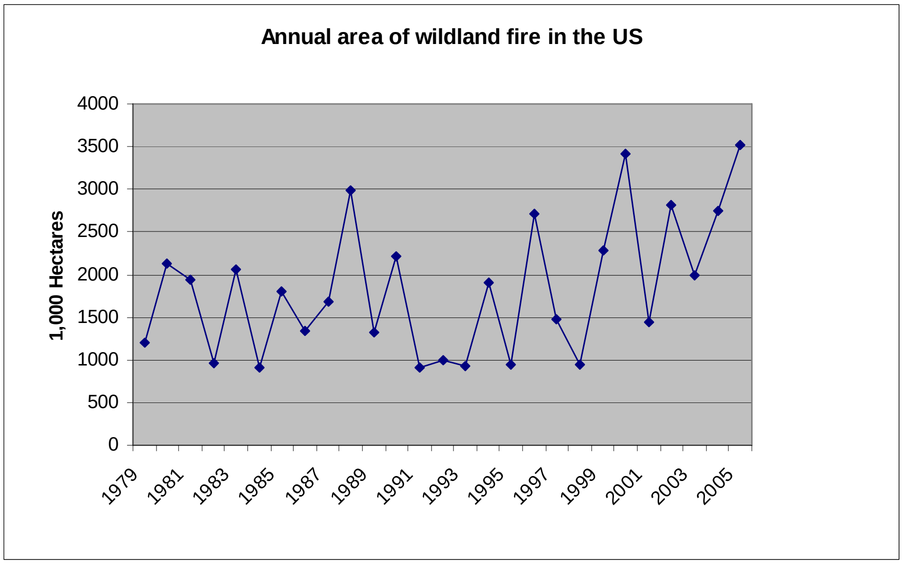

**FIGURE 2.** Historic trend in forest fires for the U.S.

## FOREST PESTS

Forest insects and pathogens are biotic disturbance agents that can be either beneficial or detrimental to forests. While they play critical roles in forest ecosystems, they can be devastating when populations reach high levels. Outbreaks can lead to damaging levels of defoliation or mortality under suitable climatic and site conditions.

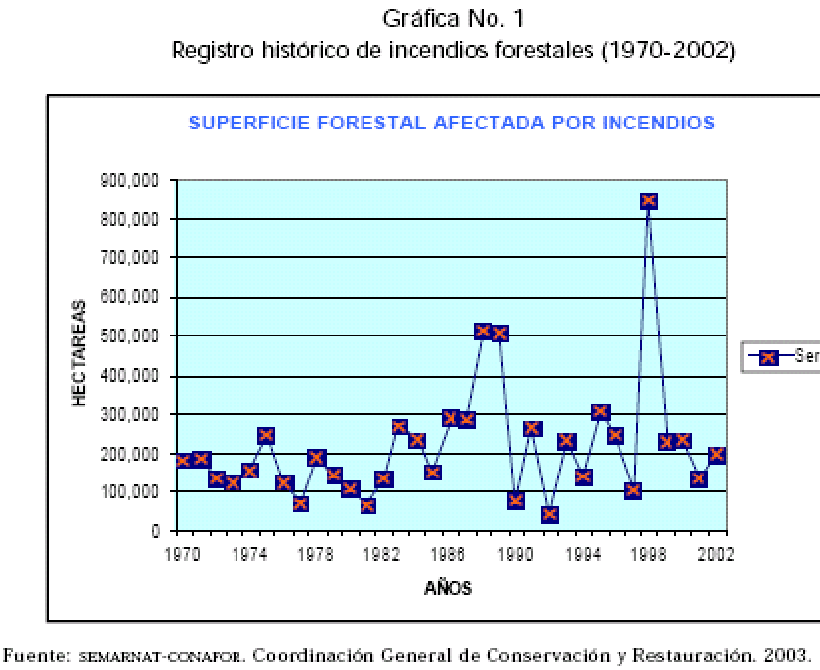

**FIGURE 3**. Historic trend in forest fires for Mexico[4].

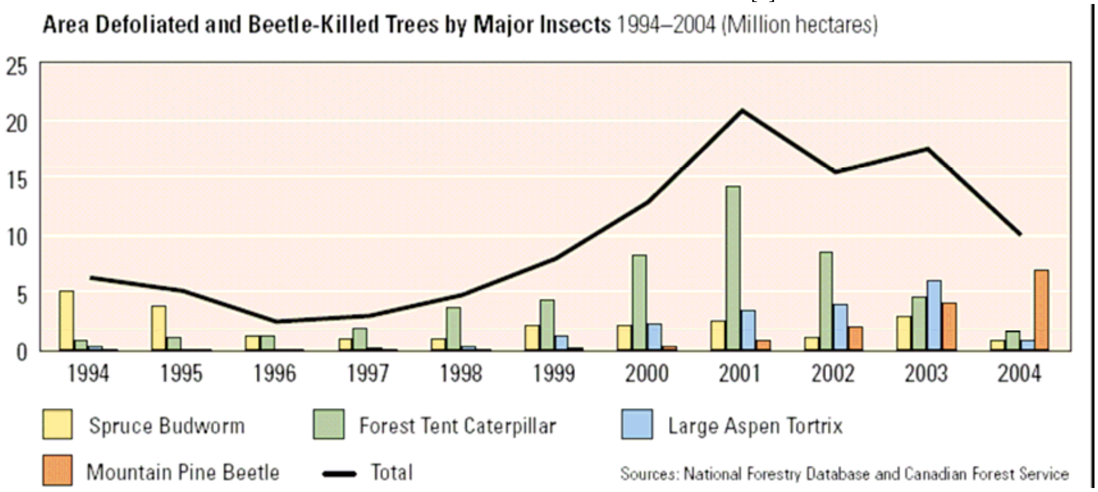

**FIGURE 4**. Historic trends in forest pest activity in Canada[3].

## Canada

Historic trends in forest pests for Canada are presented in Fig. 4[3]. The spruce budworm (*Choristoneura fumiferana*) is the most damaging insect pest of spruce and fir species in Canada. In 2004, a total of 755,325 ha were defoliated by this insect, the lowest level in the past 10 years and significantly lower than in peak years which have reached 20 million ha affected. Damage by the western spruce budworm (*C. occidentalis*), a significant pest of Douglas fir in British Columbia, has increased steadily from 123,638 ha defoliated in 2001 to 624,000 ha defoliated in 2004. Defoliation caused by the forest tent caterpillar (*Malacosoma disstria*) has decreased in recent years from a peak of nearly 15 million ha in

2001. The mountain pine beetle (*Dendroctonus ponderosae*) has caused increasing levels of mortality in British Columbia. The outbreak is the largest ever seen in North America with more than 8.7 million ha affected by 2005[3]. Approximately 450 million $m^3$ of pine have been killed.

## U.S.

Historic trends in forest pest activity in the U.S. are presented in Fig. 5. Since 2003, mountain pine beetle (*D. ponderosae*) outbreaks have been increasing in area throughout the western U.S. following several years of drought. Lodgepole pine forests have been affected the most. Southern pine beetle (*D. frontalis*) populations remain at low levels since 2003. Treatment strategies now focus on prevention and restoration. Alaska experienced a large outbreak of spruce beetle (*D. rufipennis*) in the 1990s, with mortality levels exceeding 90% in many areas. Recently, favorable weather conditions (mild winters and warm summers) led to increasing populations in Arizona, Colorado, Montana, Utah, and Wyoming. Since its introduction in 1869, the gypsy moth (*Lymantria dispar*) has spread to 17 states and the District of Columbia. The current area infested is 25% of the total susceptible area. Current management strategy focuses on slowing the spread along the advancing front of the infestation. In recent years, the effect of the biocontrol fungus, *Entomophaga maimaiga*, is evident.

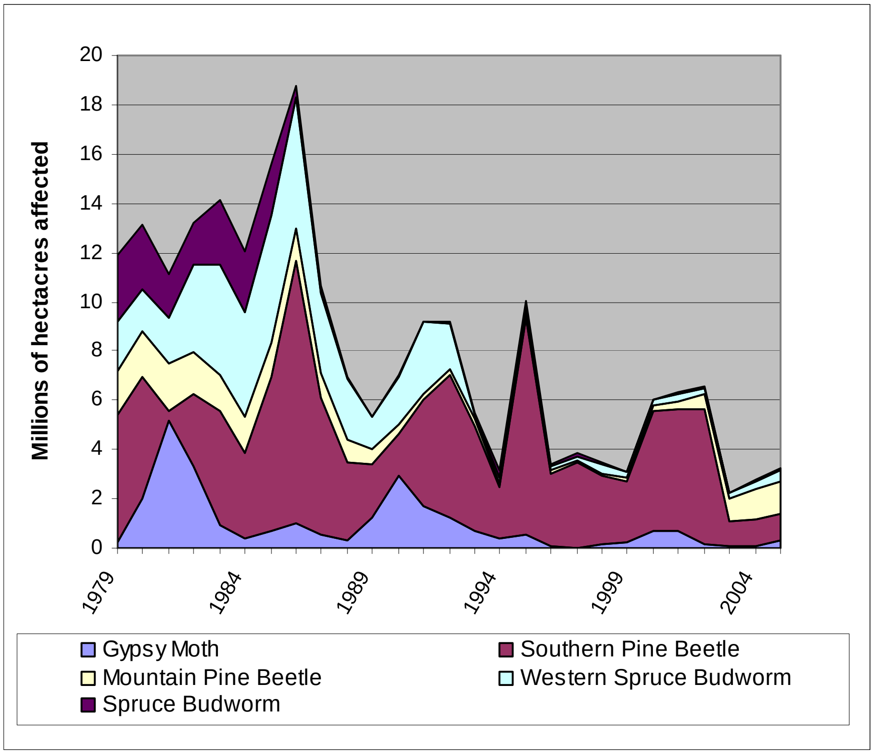

**FIGURE 5.** Historic trends in forest pest activity in the U.S.

In addition to the gypsy moth, several other invasive forest pests are threatening forests in the U.S. The hemlock wooly adelgid (*Adelges tsugae*), a native of Asia, continues to spread in eastern hemlock forests. Since its introduction in 1924, it has spread to hemlock forests from southeastern Maine to northeastern Georgia and west to eastern Tennessee. Biological control agents have been released in an attempt to control populations. The emerald ash borer (*Agrilus planipennis*), also a native of Asia, was first reported killing ash trees in the Detroit and Windsor areas of Michigan in 2002. Since then, infestations have been found throughout lower Michigan and neighboring areas in Ontario (Canada), northwest Ohio, and northern Indiana. Infestations were also recently found in the Chicago area. The European woodwasp (*Sirex noctilio*) has recently been found infesting pine trees in New York State and Ontario. Introductions of this insect into other countries have resulted in significant mortality levels in pine plantations. Monterey, lodgepole, ponderosa, jack, and most species of southern pines (especially loblolly) are known to be susceptible. The susceptibility of other North American conifers is not known. A new disease called "sudden oak death" (caused by *Phytophthora ramorum*) is killing thousands of tanoak (*Lithocarpus densiflorus*), coast live oak (*Quercus agrifolia*), and California black oak (*Q. kelloggii*) in coastal areas of California. An isolated infestation, discovered in Oregon, is being treated with goal of eradication. National surveys of oak forests have not found infestations outside California and Oregon.

## Mexico

Historic trends in forest pest activity in Mexico are presented in Fig. 6[4]. Recent bark beetle activity includes Douglas-fir beetle (*D. pseudotsugae*) in Durango; roundheaded pine beetle (*D. adjunctus*) in Chihuahua and Oaxaca; southern pine beetle (*D. frontalis*) in Guerero, Oaxaca, and Chiapas; and Mexican pine beetle (*D. mexicanus*) throughout central Mexico. The Mexican pine beetle is the bark beetle with the broadest distribution in Mexico, affecting 3,000 ha in eight states in 2004. Defoliators in Mexico include *Lophocampa alternata* in Puebla and Chihuahua, *Zadiprion falsus* in Durango and Jalisco, and *Pterophylla beltrani* in Tamaulipas. The infestation of *P. beltrani* scaled up from 200 ha in 2004 to 1700 ha in 2005, affecting mostly mixed oak vegetation types. A survey of oak mortality in central Mexico revealed the presence of *Phytophthora cinnamomi*, an exotic forest pathogen. Confirmed locations include Arrayanal and Colima in 2001, Tierra Colorada and Jalisco in 2004, and Tecoanapa and Guerrero in 2005. The pink hibiscus mealy bug, *Maconellicoccus hirsutus*, was first reported in Mexico affecting teak (*Tectona grandis*) plantations in January 2004. It is distributed in up to 10,000 ha in Valle de Banderas, Nayarit, and adjacent Jalisco State. This insect also affects mango, guava, soursop, ornamental shrubs, and at least 38 other wild plants species. Teak blight, an exotic disease to Mexico, was detected in December 2004. This disease is also found in Costa Rica, Nicaragua, El Salvador, Belize, Honduras, Guatemala, and Panama. The causal agent is the fungus *Olivea tectonae*, a parasitic disease of teak widely distributed in Asia. This disease may cause serious losses in nursery production. The presence of this disease in young plantations may cause growth losses up to 30%.

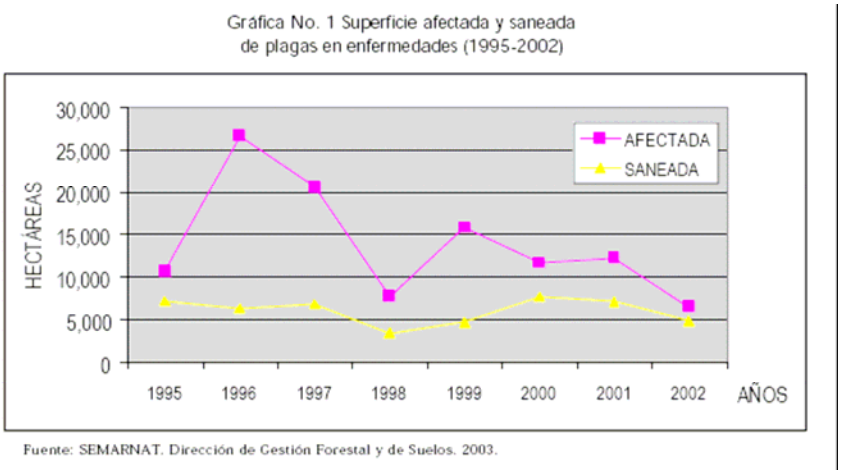

**FIGURE 6**. History of forest pest activity in Mexico.

## AIR POLLUTION

Air pollutants, including sulfur, nitrogen, and tropospheric ozone, can have significant cumulative effects on forests. Canada and the U.S. have cooperated in monitoring air pollutant deposition, concentrations, and effects under the 1991 Canada – U.S. Air Quality Agreement[5]. Spatial distribution of wet sulfate and wet nitrate deposition is presented in Figs. 7 and 8, respectively. Canada has also recently calculated critical loads exceedances of acidifying compounds for forest soils in eastern provinces (Fig. 9)[6]. For the U.S., cumulative distribution functions and frequency distributions were used to estimate the percent forest by region of the country exposed to specific levels of air pollution[7]. In the North and South, approximately 50% of the forests were exposed to sulfate deposition of more than 15 kg/ha/year for 1994–2000 (Fig. 10) compared to the Pacific Coast and Rocky Mountain regions, where approximately 50% of the forests received less than 2 kg/ha/year. Nitrate deposition was highest in the North where approximately 50% of the forests received an annual average input of more than 13 kg/ha/year. The North and South regions experienced the highest ammonium deposition rates. Ozone concentrations were relatively high across much of the South with only 10% of the forests exposed to ozone index concentrations of less than 6 ppm-h/year. Although most of the Pacific Coast region forests were exposed to relatively low ozone index, 10% of the forested area experienced exposure between 41.2 and 117.8 ppm-h/year.

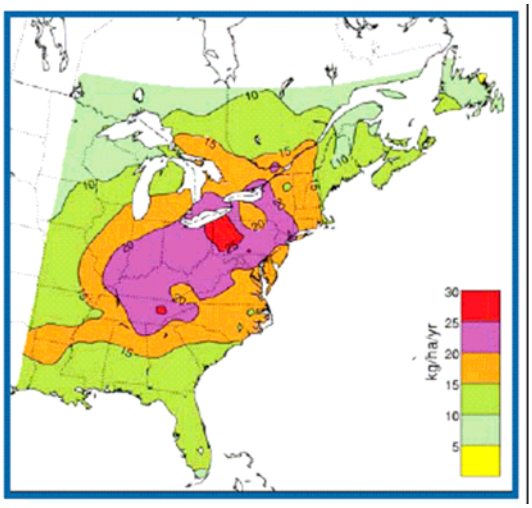

**FIGURE 7**. Spatial distribution of wet sulfate deposition (kg/ha/year) in eastern North America, 1996–2001.

## FOREST HEALTH INDICATORS

In the U.S., the Forest Health Monitoring (http://fhm.fs.fed.us) and the Forest Inventory and Analysis (http://fia.fs.fed.us) programs monitor a suite of forest health indicators to determine the effects of air pollution and other stressors.

### Crown Conditions

Crown dieback and foliar transparency measurements were used to calculate a crown index[8]. Overall, less than 15% of the basal area was associated with unhealthy crowns. Ecoregion sections having greater than 10% average basal area associated with unhealthy crowns were mostly located in the Interior West.

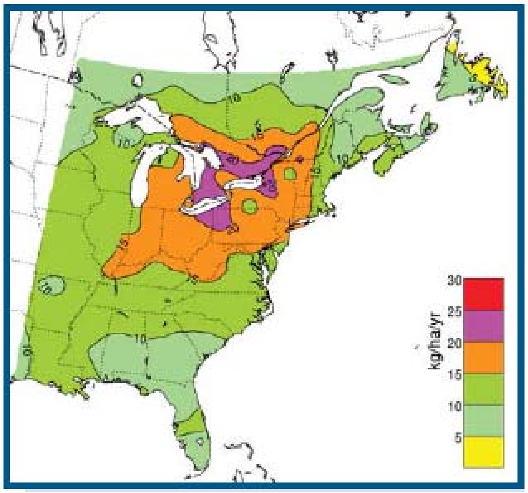

**FIGURE 8**. Spatial distribution of wet nitrate deposition (kg/ha/year) in eastern North America, 1996–2001.

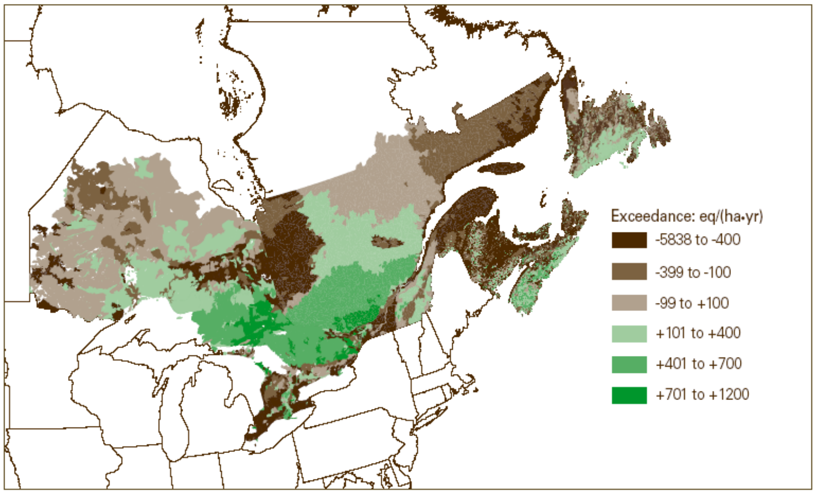

**FIGURE 9.** Critical load exceedance of acidifying compounds in Canada.

## Tree Mortality

Tree mortality on plots has been assessed using two indices: MRATIO, ratio of annual mortality volume to annual gross growth volume; and DD/LD, the ratio of the average dead tree diameter to the average live tree diameter[8]. The highest rates of mortality occurred in Idaho and western Washington.

## Soil Condition

Forest soils are critical components of forest ecosystems. The soil condition indicator collects information on physical and chemical properties of soil on measured plots[9]. The mean soil pH value for measured plots is 4.8, with acidic tendency of soil pH most clear east of the Mississippi River. The southeastern U.S. tended to have the greater proportion of forest soils with low effective cation exchange capacity levels. Total

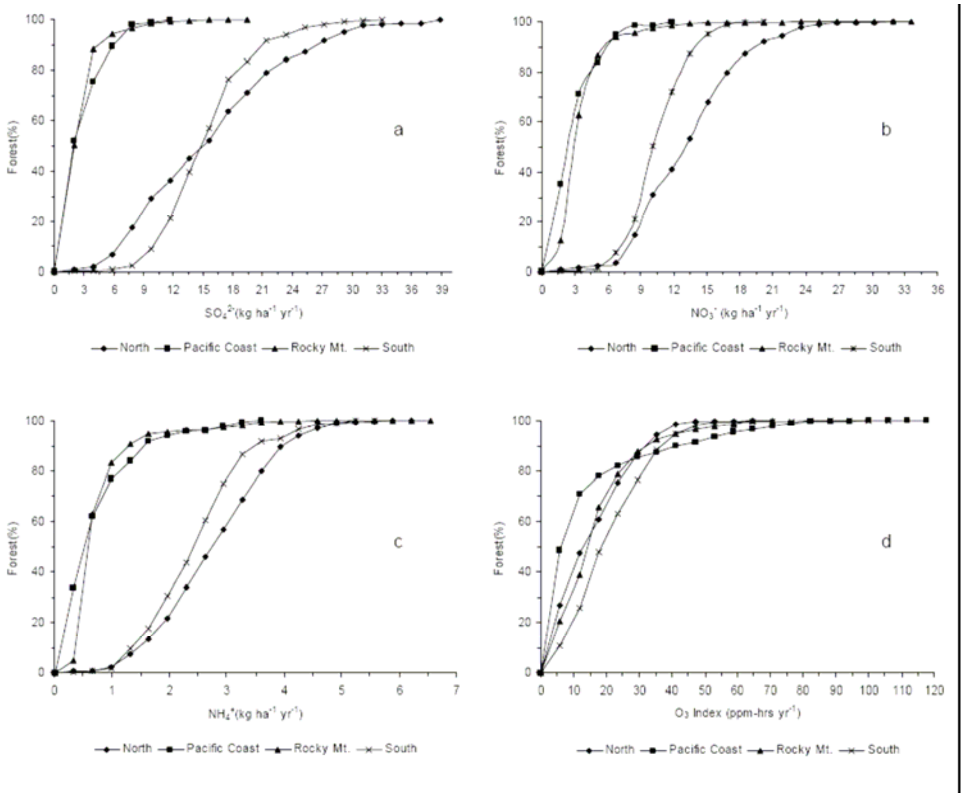

**FIGURE 10**. Proportion of forests affected by air pollutants in the U.S.

soil carbon content is generally the highest in the northeastern and northern U.S. where decay rates are very low.

## Ozone Biomonitoring

The effects of ozone on forest ecosystems are monitored by assessing damage to ozone-sensitive species on ozone biomonitoring sites located in forests throughout the U.S. The severity of foliar injury is assessed according to an injury score: 0–4.9 for no or minute injury, 5–15 for light to moderate injury, 15–25 for moderate to severe injury, and greater than 25 for severe injury. Spatial interpolations of plot injury scores for the period from 1999–2002 show that the highest foliar injury occurred in the mid-Atlantic and the southeast, with significant injury recorded in southern California.

## Lichens

The FHM and FIA programs also monitor effects of air pollution on lichen communities. Biotic indices have been developed based on lichen community data along air pollution and climate gradients. For example, spatial interpolation of lichen index scores across Washington and Oregon show a decrease of air pollution–sensitive lichens near major metropolitan areas[10].

## FUTURE CHALLENGES

In the future, we hope to enhance timely detection, analysis, and reporting of adverse changes in forest health to facilitate effective management responses. To increase our understanding of the adverse changes in forest health, we will expand our evaluations of the extent, severity, and dynamics of forest stressors. Our continued development and enhancement of national and regional risk assessments will promote development of more effective prevention strategies.
